# Recombinant BMP9 Reinforces Gut Vascular Barrier in Experimental Colitis

**DOI:** 10.3390/biomedicines14020288

**Published:** 2026-01-28

**Authors:** Shan Li, Xingyue Zhou, Yili Wang, Bingyue Yao, Siyuan Zhu, Ritian Lin, Qinjuan Sun, Jinlai Lu, Miao Hu, Wei Wang, Lan Zhong

**Affiliations:** 1 Department of Gastroenterology, Shanghai East Hospital, School of Medicine, Tongji University, Shanghai 200120, China; lishanleo@163.com (S.L.); 2432408@tongji.edu.cn (X.Z.); 2231125@tongji.edu.cn (B.Y.); 18821213132@163.com (S.Z.); ritianlin@126.com (R.L.); maomao.277@163.com (Q.S.); lujinlai1982@163.com (J.L.); hongmo123@126.com (M.H.); weiwang138@163.com (W.W.); 2 School of Medicine, Tongji University, Shanghai 200092, China; 2153620@mail.tongji.edu.cn

**Keywords:** refractory ulcerative colitis, bone morphogenetic protein 9 (BMP9), gut vascular barrier (GVB)

## Abstract

**Background:** Refractory ulcerative colitis (rUC) represents a critical therapeutic challenge, with emerging evidence implicating gut vascular barrier (GVB) dysfunction in disease persistence. We investigated whether dysregulation of the endothelial BMP9-ALK1 signaling axis—a pathway not previously studied in UC—is associated with GVB impairment and treatment resistance, and explored its therapeutic potential. **Methods:** Serum BMP9 and mucosal ALK1 levels were compared across rUC, non-rUC, and healthy cohorts. The therapeutic efficacy of BMP9 was evaluated in DSS-induced murine colitis by examining vascular permeability, histopathology, and inflammatory markers, while mechanistic roles were investigated using human intestinal microvascular endothelial cells. **Results:** Serum BMP9 levels were significantly reduced in rUC versus non-rUC patients, inversely correlating with post-treatment disease severity (Modified Mayo Score: r = −0.471, 95% CI: −0.618 to −0.293, *p* < 0.001; UCEIS: r = −0.495, 95% CI: −0.637 to −0.321, *p* < 0.001). Stratified analyses confirmed that BMP9 deficiency was associated with treatment-refractory status independent of baseline disease severity. Intestinal ALK1 was downregulated in rUC mucosa. In murine DSS-colitis, BMP9 attenuated disease severity, colon shortening, histopathological damage, inflammatory cytokines, and early pro-fibrotic markers (Col1a1, Col3a1, α-SMA). BMP9 activated SMAD1, restored VE-cadherin, and reduced hyperpermeability (FITC-dextran leakage decreased from 10.2-fold to 2.1-fold, *p* < 0.001). In vitro, BMP9 inhibited TNF-α-induced neutrophil migration and enhanced endothelial tube stability via ALK1. **Conclusions:** Dysregulated BMP9-ALK1 signaling may contribute to GVB dysfunction in UC. BMP9 supplementation attenuates vascular leakage and inflammation in experimental colitis, identifying a potential therapeutic target warranting further investigation.

## 1. Introduction

Ulcerative colitis (UC), a chronic inflammatory bowel disease characterized by mucosal inflammation of the colon [1], manifests clinically as recurrent diarrhea, abdominal pain, hematochezia, and systemic complications, significantly impairing quality of life and elevating colorectal cancer risk by approximately 2.4-fold [2]. Notably, 25–40% of UC patients progress to refractory UC (rUC), defined by inadequate response to two or more classes of licensed advanced therapies, thereby representing a critical unmet clinical need [3].

While gut microbiota dysbiosis and genetic factors have been implicated in rUC pathogenesis [4], these elements alone cannot fully explain the heterogeneity observed in rUC patients. Recent studies have highlighted the gut vascular barrier (GVB) as a potential key player in rUC progression. The GVB is a specialized endothelial-pericyte structure that regulates nutrient exchange and immune cell trafficking in the intestinal mucosa [5]. Clinical evidence strongly supports its involvement: in vedolizumab-resistant patients, activation of the α4β1/VCAM-1 pathway on GVB endothelial cells facilitates abnormal lymphocyte homing to inflamed mucosa [6]. Furthermore, transcriptomic analyses of anti-TNFα non-responders reveal distinct signatures of endothelial hyperproliferation [7], suggesting that GVB dysfunction may sustain inflammation through structural vascular changes independent of immune mechanisms. These findings collectively position GVB impairment as both a marker of disease severity and a promising therapeutic target in rUC. However, the specific molecular mechanisms underlying GVB dysfunction in rUC remain poorly understood. Current therapeutic strategies for rUC primarily focus on modulating immune responses [8], with limited attention to the molecular regulation of vascular barrier function. Given the emerging importance of GVB dysfunction in treatment-resistant UC, identifying endothelial-specific regulators may reveal new therapeutic targets beyond conventional immunomodulatory approaches.

The bone morphogenetic protein 9 (BMP9)-activin receptor-like kinase 1 (ALK1) signaling axis is a well-established regulator of vascular quiescence, functioning to inhibit endothelial proliferation, stabilize intercellular junctions, and maintain barrier integrity [9]. BMP9, predominantly secreted by hepatic stellate cells, circulates in the bloodstream and acts as the primary ligand for ALK1, an endothelial-specific receptor [10]. Although BMP10, the closest structural paralogue of BMP9 (65% amino acid identity), also activates ALK1, it is predominantly cardiac-derived and regulates ventricular development rather than systemic vascular homeostasis [11]. Several observations prompted us to investigate a potential role for this pathway in intestinal vascular dysfunction. First, ALK1 is preferentially expressed in colonic vascular endothelium (1.6-fold higher than epithelial cells), suggesting a predominant role in intestinal vascular regulation [12]. Second, our previous work demonstrated that BMP9 suppresses hepatic macrophage infiltration and ameliorates liver inflammation [13], suggesting broader anti-inflammatory properties that may extend to intestinal inflammation. Third, BMP9 deficiency is associated with vascular destabilization in multiple organ systems, including spontaneous gastrointestinal vascular malformations in Bmp9^−/−^ mice [14]. Collectively, these findings suggest that impaired BMP9-ALK1 signaling may contribute to the vascular abnormalities characteristic of rUC.

Based on the above findings, we hypothesize that dysregulation of the BMP9-ALK1 axis in the colonic microvasculature may represent a potential mechanism underlying GVB dysfunction in rUC. To address this hypothesis, we first examined whether serum BMP9 levels and mucosal ALK1 expression are altered in rUC patients compared to non-refractory UC and healthy controls. We then evaluated whether BMP9 supplementation attenuates GVB dysfunction in a murine model of experimental colitis, and dissected the underlying molecular mechanisms using in vitro endothelial cell systems. Our findings reveal a previously unrecognized association between BMP9-ALK1 dysregulation and intestinal vascular barrier impairment, providing new insights into rUC pathogenesis and identifying a potential therapeutic target.

## 2. Materials and Methods

### 2.1. Subjects

This study recruited UC patients from the Department of Gastroenterology, Shanghai East Hospital, Tongji University, between February 2024 and December 2024. Eligible participants were adults (18–70 years) with active disease (Modified Mayo Score ≥ 3; endoscopic subscore ≥ 2) diagnosed per European Crohn’s and Colitis Organisation (ECCO) guidelines [15]. Exclusion criteria encompassed pregnancy, lactation, active infection, malignancy, severe comorbidities (e.g., Child-Pugh B/C cirrhosis, heart failure, renal impairment), and contraindications to colonoscopy or advanced therapies. Ongoing corticosteroid use was permitted.

Baseline serum samples and clinical data were collected pre-treatment during endoscopic assessment. Patients were retrospectively classified based on their response to ≥14 weeks of advanced therapy. The rUC cohort comprised patients who failed ≥2 classes of advanced therapies (biologics or small molecules), defined as a lack of clinical response (Mayo score reduction < 3) [16]. Conversely, the non-rUC group included patients achieving clinical response within 14 weeks of their first advanced therapy. Age- and sex-matched healthy controls were recruited from the health screening center, excluding individuals with mucosal inflammation, history of gastrointestinal disease, or a family history of IBD. Disease severity was assessed using the Modified Mayo Score (MMS; range 0–12, combining stool frequency, rectal bleeding, and physician global assessment subscores) and the Ulcerative Colitis Endoscopic Index of Severity (UCEIS; range 0–8) [17]. Paired serum and mucosal biopsy specimens were retrieved from the Shanghai East Hospital biobank (Ethics No.: 2021YS-107), with written informed consent obtained from all participants.

### 2.2. Mice

Male C57BL/6J mice (6 weeks old, 18–20 g) were obtained from GemPharma Tech (Nanjing, China) and housed under specific pathogen-free (SPF) conditions in the Tongji University Animal Facility (ambient temperature 22 ± 1 °C, 12 h light/dark cycle, ad libitum access to autoclaved feed and water). All experimental procedures were approved by the Institutional Animal Care Guidelines authorized by the Tongji University Animal Research Ethics Committee (Approval No.: TJBB05424102). Mice aged 8–10 weeks were utilized for subsequent experiments to ensure physiological maturity and standardized baseline characteristics.

### 2.3. Acute Colitis Induction and BMP9 Intervention

The dextran sulfate sodium (DSS)-induced colitis model was established in 8-week-old male C57BL/6J mice by administering 3% (*w*/*v*) DSS (MW 36–50 kDa; Yeasen, Shanghai, China) dissolved in autoclaved drinking water ad libitum for 7 consecutive days, followed by a 3-day recovery period with normal drinking water. Disease activity was monitored daily by assessing body weight change (%), stool consistency (0: formed pellets; 4: liquid diarrhea), and fecal occult blood (detected using Hemoccult tests). For histological assessment, mice were sacrificed on day 10. Recombinant murine BMP9 (MCE, HY-P700530) was administered via intraperitoneal injection (200 ng/mouse/day) from day 0 to day 7, concurrent with DSS induction. The dosage was determined based on our previously published work demonstrating efficacy and safety at this concentration [13]. Control mice received an equivalent volume of PBS. High-resolution mini-endoscopy of colitic mice was performed using an endoscope (Karl Storz, Tuttlingen, Germany) on day 10.

### 2.4. Vascular Permeability Assessment

Assessment of small-molecule leakage: DSS-induced colitis mice fasted for 12 h on day 8 received 500 mg/kg FITC-dextran (4 kDa; Beyotime, Haimen, China) by orogastric gavage. 60 min post-administration, serum was isolated from retro-orbital blood samples after centrifugation. Fluorometric quantification (492/520 nm excitation/emission) was performed using calibration curves, with saline-gavaged mice providing background reference. Intestinal-to-systemic permeability (paracellular barrier function) was measured by quantifying serum FITC-dextran fluorescence following oral gavage, reflecting cumulative intestinal barrier integrity.

Assessment of macromolecular extravasation: Mice received a tail vein injection of 0.2 mL 0.5% Evans blue (Sigma, Saint Louis, MO, USA). After 2 h of circulation, systemic perfusion with PBS was performed to clear intravascular dye. Excised colonic tissues were homogenized in N,N-dimethylformamide (4 μL/mg tissue), incubated at 60 °C for 24 h, and centrifuged. The supernatant was collected, and dye concentration in the supernatant was measured spectrophotometrically at 620 nm and normalized to tissue weight (μg/g). Local colonic vascular leakage was assessed by Evans blue extravasation specifically in excised colonic tissue segments (distal colon, standardized 3 cm segment proximal to the rectum), providing direct measurement of GVB dysfunction at the primary site of DSS-induced injury.

### 2.5. Cell Culture

Human immortalized intestinal microvascular endothelial cells (HIMECs, Shanghai Yuchun Biotechnology, Shanghai, China, #CM2049) at passages 5–8 were maintained in endothelial cell medium (ScienCell, Carlsbad, CA, USA, #1001) supplemented with 5% fetal bovine serum, 1% endothelial cell growth supplement, and 1% penicillin/streptomycin at 37 °C in a 5% CO_2_ humidified incubator.

### 2.6. In Vitro Tube Formation Assay

24-well plates were pre-cooled at 4 °C, and each well was coated with 20 µL of Matrigel (Corning, NY, USA, #356234), which was then polymerized at 37 °C for 1 h to form a homogeneous gel. HIMECs at 90% confluency were detached using 0.05% trypsin-EDTA, resuspended in endothelial cell medium, and seeded onto the Matrigel-coated wells at a density of 1.5 × 10^5^ cells/well (500 µL/well). Immediately after seeding, HIMECs were treated with the following stimuli: recombinant human BMP9 (rhBMP9; 0–10 ng/mL), TNF-α (20 ng/mL; Novoprotein, Suzhou, China, #C008), and ML347 (150 nM; MCE, Monmouth Junction, NJ, USA, #HY-12274). Plates were incubated under standard culture conditions (37 °C, 5% CO_2_, 95% humidity) for 12 h. The tubular networks were monitored at 4 h intervals using an inverted phase-contrast microscope (10× magnification). Images were processed using ImageJ 1.53 (National Institutes of Health, Bethesda, MD, USA) with the Angiogenesis Analyzer plugin to quantify branch points and nodes.

### 2.7. Neutrophil Migration Assay

Neutrophils were isolated from peripheral blood of healthy donors using a Human Peripheral Blood Neutrophil Isolation Kit (Solarbio, Beijing, China) and labeled with 5 μM Calcein-AM (Yeasen Biotechnology, Shanghai, China) in PBS for 30 min at 37 °C. HIMECs were seeded and cultured in the lower chamber of a 3 μm pore-size 24-well Transwell plate (Corning Inc., Corning, NY, USA) and allowed to adhere prior to pretreatment. Cells were pretreated with BMP9 (0, 0.1, 1, or 10 ng/mL), TNFα (20 ng/mL), or ML347 (150 nM) for 2 h.

Subsequently, 1 × 10^6^ labeled neutrophils were added to the upper chamber and allowed to migrate toward HIMECs for 1 h under standard culture conditions (37 °C, 5% CO_2_). Migrated neutrophils in the lower chamber were quantified by measuring fluorescence intensity (excitation/emission: 490/515 nm) in 10 random microscopic fields per well using an inverted fluorescence microscope, and further validated by collecting 150 μL medium from both chambers for fluorescence detection via a microplate reader. Migration rate (%) = Lower chamber fluorescence/Total fluorescence (Upper + Lower) × 100%.

### 2.8. Enzyme-Linked Immunosorbent Assay (ELISA)

Serum concentrations of human BMPs were quantified using commercial enzyme-linked immunosorbent assay (ELISA) kits (BiotechWell, Shanghai, China). ALK1 protein levels in murine intestinal tissues were analyzed using species-specific ELISA kits (Coibo Bio, Shanghai, China). Serum samples were centrifuged (3000× *g*, 10 min) to remove cellular debris, and supernatants were aliquoted and stored at −80 °C until analysis. Murine intestinal tissues (approximately 30 mg) were mechanically homogenized in ice-cold RIPA lysis buffer (Beyotime Biotechnology, #P1010) containing protease and phosphatase inhibitors. Tissue lysates were clarified by centrifugation (12,000× *g*, 15 min, 4 °C) prior to analysis. All assays were performed in triplicate according to manufacturer specifications, with absorbance measured at 450 nm using an Infinite 200 PRO microplate reader (Tecan, Männedorf, Switzerland).

### 2.9. Hematoxylin and Eosin (H&E) Staining

Formalin-fixed intestinal tissues from mice and humans were embedded in paraffin and sectioned at 4 μm thickness. Sections were deparaffinized in an eco-friendly dewaxing solution (3 × 10 min), rehydrated through a graded ethanol series (100%, 95%, 80%), and stained with hematoxylin (5 min) followed by eosin (1 min). After dehydration in ethanol and xylene, slides were mounted with neutral resin.

### 2.10. Multiplex Fluorescent Tyramide Signal Amplification (TSA) Staining

Paraffin-embedded intestinal tissues from mice and humans were fixed with 4% paraformaldehyde, deparaffinized using an eco-friendly solvent, rehydrated through graded ethanol, and subjected to antigen retrieval in citrate buffer (pH 6.0) by high-pressure heating. After cooling, hydrophobic barriers were drawn around tissues to localize reagents, and endogenous peroxidase activity was blocked with 3% H_2_O_2_. Sections were blocked with 3% BSA and incubated with primary antibodies at 4 °C overnight: goat anti-CD31 (1:100, Servicebio, Wuhan, China) and rabbit anti-VE-cadherin (1:200, Affinity, Cincinnati, OH, USA) for mouse tissues; mouse anti-CD31 (1:500, Servicebio) and rabbit anti-VE-cadherin (1:200, Affinity) for human tissues. HRP-conjugated secondary antibodies, diluted appropriately, were applied for 1 h at room temperature, followed by incubation with tyramide-conjugated fluorophores (TSA reagent, 10 min). Sequential stripping of antibodies was performed via microwave heating in citrate buffer (validated by no-signal controls), enabling iterative staining cycles for additional targets.

### 2.11. Quantitative Real-Time PCR

Total RNA was isolated using the RNAeasy™ Animal RNA Isolation Kit with Spin Column (Beyotime). cDNA was synthesized from RNA with PrimeScript RT Master Mix (Takara, Shiga, Japan), which includes genomic DNA removal. qPCR reactions were performed in triplicate on a QuantStudio 7 Flex System (Applied Biosystems, Carlsbad, CA, USA) using TB Green Premix Ex Taq™ (Takara) and target-specific primers (Supplementary Table S1), followed by melt curve analysis. Data were normalized to *GAPDH* expression and analyzed via the ΔΔCt method. ALK1 expression was assessed at multiple levels: (1) mRNA expression was quantified by RT-qPCR from colonic mucosal biopsies (normalized to GAPDH); (2) protein levels were determined by ELISA in tissue homogenates.

### 2.12. Western Blot Analysis

Tissues or cells were lysed in RIPA buffer supplemented with protease/phosphatase inhibitors, and protein concentrations were determined by BCA assay. Equal amounts of protein (20–50 μg) were resolved on SDS-PAGE gels (8–12%) and transferred to PVDF membranes. Membranes were blocked with QuickBlock™ Blocking Buffer (Beyotime) for 30 min at room temperature, followed by overnight incubation at 4 °C with primary antibodies: VE-cadherin (1:1000, abcam, Cambridge, UK, ab205336), CCL2 (1:1000, CST, Danvers, MA, USA, #2029), SMAD1 (1:1000, CST, #6944), p-SMAD1 (1:1000, abcam, ab214423), TGFβ (1:1000, Proteintech, Rosemont, IL, USA, 21898-1-AP), α-SMA (1:500, Invitrogen, Carlsbad, CA, USA, MA5-11547), and β-actin (1:50,000, Abclonal, Woburn, MA, USA, AC026). After washing, membranes were incubated with H + L anti-rabbit/mouse (1:50,000, Invitrogen, 31430/31460) secondary antibodies for 1 h at room temperature. Protein bands were visualized using enhanced chemiluminescence (ECL, Tanon 5200 Chemiluminescence System, Shanghai, China). For reprobing, membranes were stripped with stripping buffer, re-blocked, and re-probed with subsequent antibodies. β-actin served as the loading control, and data were normalized to β-actin expression. Images were processed using ImageJ 1.53.

### 2.13. Bioinformatics Methodology

Total RNA for sequencing was isolated using TRIzol reagent (Invitrogen, USA), followed by purity assessment (A260/A280 > 1.8) and integrity verification (RIN > 7.0) via NanoDrop ND-1000 spectrophotometry (Wilmington, DE, USA) and Agilent 2100 Bioanalyzer (Santa Clara, CA, USA). Polyadenylated mRNA was enriched using Dynabeads Oligo(dT)25 beads (Thermo Fisher, Waltham, MA, USA) and fragmented at 94 °C for 5–7 min (NEBNext^®^ Magnesium RNA Fragmentation Module). First- and second-strand cDNA synthesis was performed using SuperScript™ II Reverse Transcriptase and E. coli DNA polymerase I/RNase H (NEB, Ipswich, MA, USA), incorporating dUTP for strand specificity. Libraries were prepared via end-repair, A-tailing, adapter ligation, and size selection (300 ± 50 bp inserts) with AMPure XP beads (Beckman Coulter, Brea, CA, USA). PCR amplification (8 cycles) and UDG digestion generated strand-specific libraries, sequenced as 150 bp paired-end reads on an Illumina NovaSeq 6000 (LC-Bio, Hangzhou, China).

Raw sequencing data were processed using fastp (v0.23.2) to remove adapter-contaminated reads, low-quality bases (Q < 20), and ambiguous nucleotides (>5%). Quality-filtered reads were aligned to the human reference genome GRCh38 using HISAT2 (v2.2.1). Transcript assembly and FPKM-based quantification were performed via StringTie, followed by differential expression analysis with edgeR (FDR-adjusted *p* < 0.05, |log2(fold change)| > 1). Functional enrichment of differentially expressed genes was conducted using DAVID, focusing on Kyoto Encyclopedia of Genes and Genomes (KEGG) pathways.

### 2.14. Statistical Analysis

Data were analyzed using SAS version 9.4 (SAS Institute, Cary, NC, USA) and GraphPad Prism 9.0 (GraphPad Software, Boston, MA, USA). Continuous variables are expressed as mean ± standard deviation (SD) for normally distributed data or median (interquartile range) for non-normally distributed data; categorical variables are presented as frequency (percentage). Normality was assessed using Shapiro–Wilk tests. For two-group comparisons, independent *t*-tests or Mann–Whitney U tests were used as appropriate. For three-group comparisons, one-way ANOVA with Tukey’s post hoc test or Kruskal–Wallis H test with Dunn’s correction was applied. Two-factor repeated-measures analysis was performed using mixed-design ANOVA with Greenhouse-Geisser correction when sphericity (assessed by Mauchly’s test) was violated. Comparisons between groups used chi-square tests, continuity-corrected chi-square tests, or Fisher’s exact probability tests. Spearman’s rank correlation coefficient was used to evaluate associations between variables. Statistical significance was defined as *p* < 0.05 (two-tailed). To isolate the impact of treatment refractoriness from baseline disease severity, stratified analyses were performed based on Modified Mayo Score categories: mild (3–5), moderate (6–10), and severe (11–12). Within each stratum, serum BMP9 levels were compared between rUC and non-rUC groups using independent *t*-tests.

## 3. Results

### 3.1. Pathological Vascular Alterations in rUC

A total of 145 participants were enrolled in this study, including 50 healthy controls, 47 non-rUC patients, and 48 rUC patients. Baseline characteristics are summarized in Table 1. The three groups were comparable in age (*p* = 0.86), sex distribution (*p* = 0.89), and smoking status (*p* = 0.29). As expected, UC patients had significantly lower BMI [non-rUC: 20.9 (19.35, 21.9); rUC: 20.35 (19.45, 21.85)] compared to healthy controls [23.7 (21.68, 25.9)] (*p* < 0.001), reflecting the nutritional impact of active disease. Laboratory parameters including elevated CRP (non-rUC: 33.5 ± 16.6; rUC: 39.8 ± 14.3 mg/L), decreased hemoglobin, and decreased albumin were observed in UC patients compared to healthy controls (all *p* < 0.001), with no significant differences between non-rUC and rUC groups.

**Figure 1:**
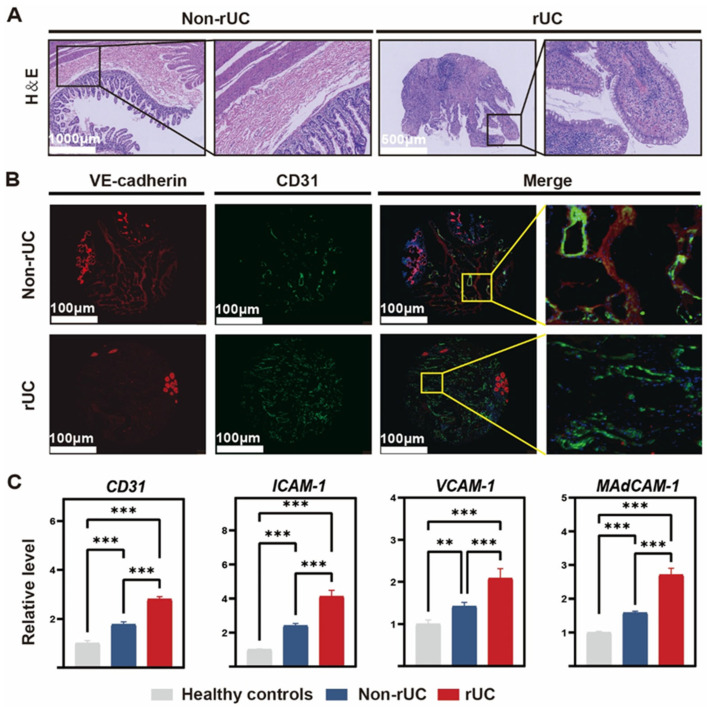
Distinct mucosal and vascular pathology in rUC compared to non-rUC and healthy controls. (**A**) Representative H&E-stained sections of colonic mucosa. (**B**) Immunofluorescence co-localization of VE-cadherin (red), CD31 (green) in colonic tissues, with DAPI nuclear counterstaining. (**C**) Relative expression levels of CD31, VCAM-1, ICAM-1, and MadCAM-1 in colonic mucosa (healthy controls, non-rUC, rUC; *n* = 4 per group). Data derived from RT-qPCR (normalized to GAPDH; mean ± SD). Statistical significance determined by one-way ANOVA with Tukey’s post hoc test: ** *p* < 0.01, *** *p* < 0.001.

We first compared intestinal mucosal pathology between rUC and non-rUC patients. Histopathological analysis revealed crypt distortion with neutrophil infiltration in rUC mucosa and significantly increased vascular density in the rUC group compared to non-rUC (Figure 1A). Immunofluorescence co-localization analysis indicated reduced expression of VE-cadherin—a critical molecule for endothelial adherens junctions—in the rUC group compared to healthy controls, suggesting impaired vascular barrier function in rUC (Figure 1B). Additionally, qPCR analysis of endothelial cell junctional and adhesion molecule transcripts (*CD31, VCAM1*, *ICAM1*, and *MadCAM1*) in colonic mucosal tissues revealed significant upregulation in rUC patients compared to both healthy controls and non-rUC patients (Figure 1C). These findings collectively indicate dysregulated vascular homeostasis in rUC.

### 3.2. BMP9/ALK1 Signaling Dysregulation in rUC

**Figure 2:**
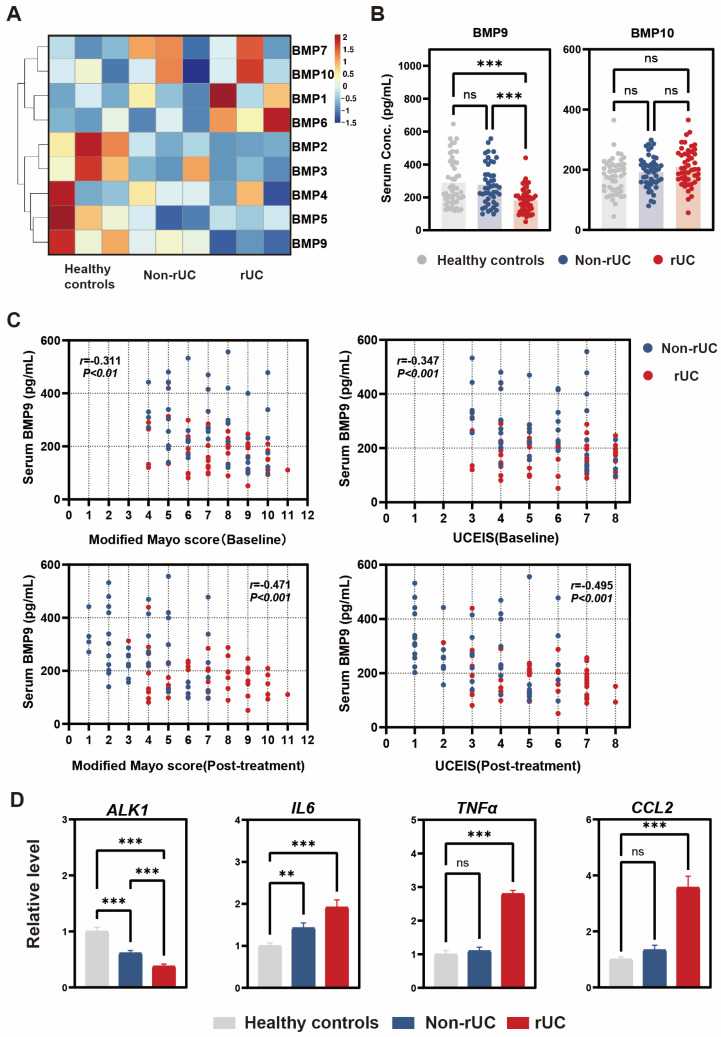
Dysregulation of BMP9/ALK1 signaling and inflammation in refractory ulcerative colitis (rUC). (**A**) Heatmap depicting serum expression levels of BMP family members in healthy controls, non-rUC, and rUC patients (*n* = 3 per group). Data were Z-score normalized and hierarchically clustered (red, high expression; blue, low expression). (**B**) Baseline serum levels of BMP9 and BMP10 in healthy controls (*n* = 50), non-rUC patients (*n* = 47), and rUC patients (*n* = 48). (**C**) Spearman correlation analyses between baseline serum BMP9 levels and clinical disease activity indices, including baseline Modified Mayo Score, baseline UCEIS, post-treatment Modified Mayo Score, and post-treatment UCEIS, in patients with UC (*n* = 95). (**D**) Colonic mucosal mRNA expression levels of ALK1, IL-6, TNF-α, and CCL2 in healthy controls, non-rUC, and rUC patients (*n* = 4 per group). Statistical annotations for (**B**,**D**): (Normalized to GAPDH; Mean ± SD; Statistical significance determined by one-way ANOVA with Tukey’s post hoc test: ** *p* < 0.01, *** *p* < 0.001, ns: not significant).

To identify regulators of gut vascular barrier (GVB) dysfunction, we screened BMP family ligands and found significantly reduced serum BMP9 levels in rUC patients versus non-rUC counterparts (Figure 2A). Notably, despite structural homology between BMP9 and BMP10 (65% amino acid sequence similarity), serum BMP10 concentrations showed no intergroup differences (*p* = 0.21; Figure 2B). Importantly, at baseline (pre-treatment), when serum BMP9 was measured, the rUC and non-rUC groups had comparable disease activity as assessed by the Modified Mayo Score ([8 (6, 9) vs. 7 (5, 8.5), *p* = 0.23] and UCEIS [6 (4, 7) vs. 5 (4, 7), *p* = 0.298; Table 1). Despite comparable baseline disease activity, serum BMP9 levels were significantly lower in rUC patients (181.42 ± 75.85 ng/mL) compared to both non-rUC patients (276.32 ± 122.77 ng/mL, *p* < 0.001) and healthy controls (289.85 ± 143.20 ng/mL, *p* < 0.001). Notably, serum BMP9 levels in non-rUC patients were similar to those in healthy controls (*p* = 0.62), suggesting that BMP9 deficiency is specifically associated with treatment-refractory status rather than UC diagnosis per se. This observation guided our subsequent mechanistic investigation of the BMP9-ALK1 axis.

Correlation analyses revealed that baseline serum BMP9 levels were modestly but significantly correlated with baseline disease activity (Modified Mayo Score: r = −0.311, 95% CI: −0.487 to −0.111, *p* < 0.01; UCEIS: r = −0.347, 95% CI: −0.517 to −0.151, *p* < 0.001). Notably, baseline BMP9 showed stronger correlations with post-treatment outcomes (Modified Mayo Score: r = −0.471, 95% CI: −0.618 to −0.293, *p* < 0.001; UCEIS: r = −0.495, 95% CI: −0.637 to −0.321, *p* < 0.001; Figure 2C). To further confirm that the association between BMP9 deficiency and treatment-refractory status was independent of baseline disease severity, we performed stratified analyses according to the Modified Mayo Score (Supplementary Table S2). Within the moderate severity stratum (Mayo 6–10), which comprised the majority of patients (*n* = 74, 78%), serum BMP9 levels remained significantly lower in rUC patients compared to non-rUC patients (172.52 ± 62.18 vs. 260.60 ± 126.40 ng/mL, *p* < 0.001). The mild severity stratum (Mayo 3–5) showed a similar trend (242.37 ± 119.36 vs. 317.44 ± 106.37 ng/mL, *p* = 0.191), although this did not reach statistical significance, likely due to the limited sample size (*n* = 20). These findings support an independent association between BMP9 deficiency and treatment-refractory status after controlling for baseline disease severity.

Quantitative RT-PCR analyses revealed significant downregulation of ALK1 mRNA expression in the intestinal mucosa of UC patients compared to healthy controls (61% of control levels; *p* < 0.001), with a further reduction in rUC cohorts (38% of non-rUC levels; *p* < 0.001). Concurrently, mucosal transcripts of proinflammatory mediators IL-6, TNF-α, and CCL2 were markedly elevated in rUC patients relative to non-rUC patients (all *p* < 0.001; Figure 2D).

### 3.3. BMP9 Supplementation Attenuates DSS-Induced Colitis via Smad Signaling

**Figure 3:**
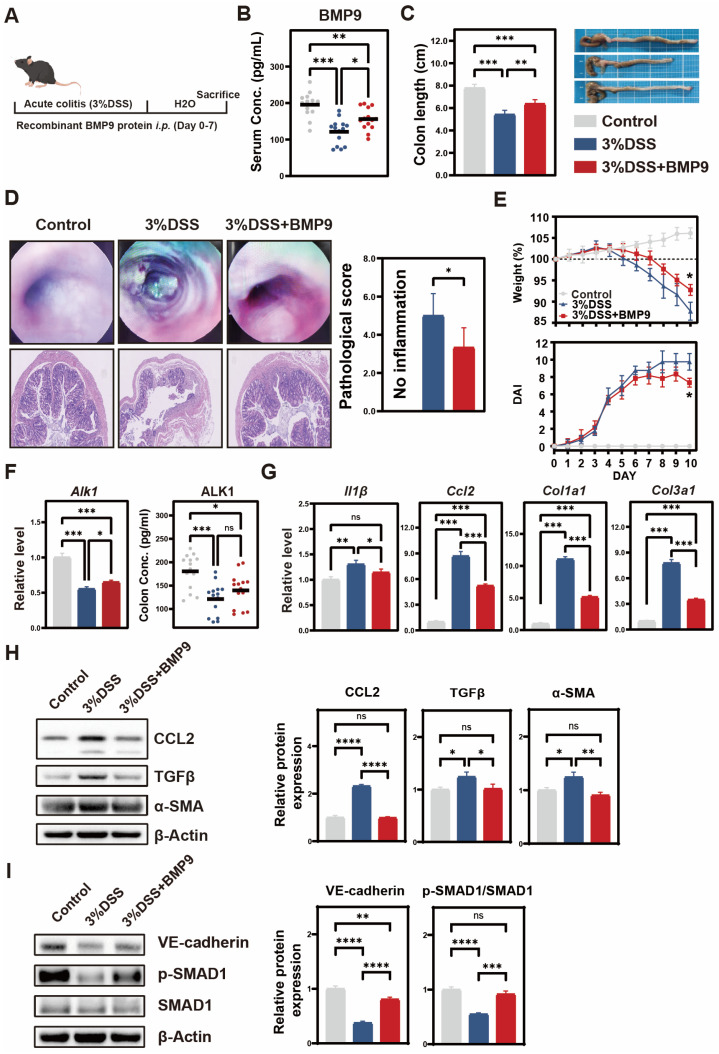
BMP9 attenuates DSS-induced colitis in mice. (**A**) Schematic of experimental design: Acute colitis was induced in C57BL/6 mice by 3% DSS in drinking water for 7 days. The BMP9 treatment group received intraperitoneal injections of recombinant murine BMP9 (200 ng/day), while the DSS group and the control group received PBS (*n* = 14 per group). (**B**) Serum BMP9(ng/mL) concentrations measured by ELISA. (**C**) Representative images of colons and quantitative analysis of colon length (cm). (**D**) Colonoscopy images (upper), H&E-stained colon sections (lower; scale bars = 100 μm), and histopathological scores. (**E**) (Upper) Dynamic body weight changes and (Lower) Disease Activity Index (DAI) scores. Data expressed as mean ± SD; * *p* < 0.05, two-way repeated measures ANOVA. (**F**) (Left) Relative Alk1 mRNA expression in colon tissues (RT-qPCR normalized to Gapdh). (Right) ALK1 protein concentrations (quantified by ELISA). Data presented as mean ± SD (* *p* < 0.05, ** *p* < 0.01, *** *p* < 0.001, **** *p* < 0.0001 ns: not significant). (**G**) Relative mRNA expression levels of IL-1β, Ccl2, Col1a1, and Col3a1 (RT-qPCR; mean ± SD). (**H**) Western blot analysis of CCL2, TGF-β, and α-SMA protein expression in colon tissues and quantitative analysis of band intensities. (**I**) Western blot detection of p-Smad1, total Smad1, and VE-cadherin proteins and quantitative analysis of band intensities.

Given the observed vascular pathology, we established a DSS-induced colitis model to determine whether analogous alterations occur in the BMP9 signaling axis (Figure 3A). DSS-treated mice exhibited hallmark disease features, including significant colon shortening (5.3 ± 0.2 cm vs. control 7.8 ± 0.3 cm; *p* < 0.001) and elevated histopathological scores (8.2 ± 1.1 vs. 1.3 ± 0.4; *p* < 0.001). Serum ELISA revealed a 38% reduction in BMP9 concentration in DSS mice (121.3 ± 34.5 ng/mL) versus healthy controls (195.6 ± 33.5 ng/mL; *p* < 0.001), mirroring the decrease observed in active UC patients (Figure 3B). BMP9 supplementation significantly improved colon length to 6.4 ± 0.3 cm (*p* < 0.01 vs. DSS group), achieving 82.1% of the length observed in controls (Figure 3C). Colonoscopy and H&E staining demonstrated a reduction in ulcerated areas and a 2.2 ± 0.7-point decrease in histological scores in BMP9-treated mice (Figure 3D). The BMP9 cohort also showed attenuated weight loss and significantly lower disease activity index (DAI) scores versus DSS controls (Figure 3E). Notably, DSS induced a significant decrease in colonic ALK1 gene expression (approximately 46%) compared with the control group (*p* < 0.001; Figure 3F), which was not rescued by BMP9 supplementation.

Despite this persistent ALK1 downregulation, BMP9 intervention significantly suppressed proinflammatory cytokine expression (IL-1β, CCL2) and early pro-fibrotic markers at mRNA (Col1a1, Col3a1) and protein (α-SMA, TGFβ) levels (Figure 3G–H). Furthermore, Western blot analysis confirmed BMP9-mediated activation of the Smad pathway (increased p-Smad1/Smad1 ratio) and restoration of VE-cadherin expression in colonic tissues (Figure 3I). Upon quantitative analysis, the p-SMAD1/SMAD1 ratio in the DSS + BMP9 group was partially restored but did not fully return to control levels (DSS + BMP9: 93.6 ± 6.1% of control vs. DSS alone: 54.8 ± 2% of control; *p* < 0.001).

### 3.4. BMP9 Restores Endothelial Barrier Function via VE-Cadherin Stabilization

**Figure 4:**
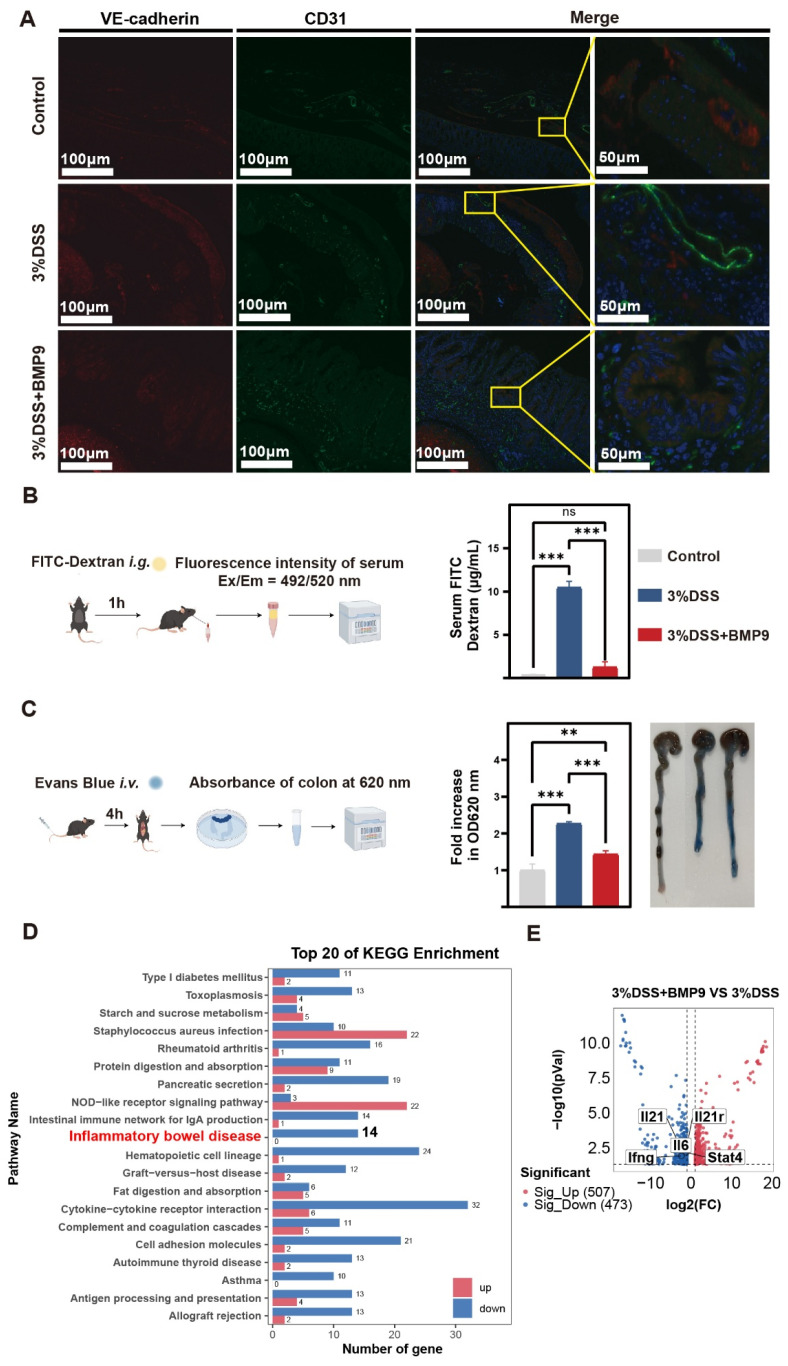
BMP9 restores intestinal vascular barrier integrity in DSS-induced colitis. (**A**) Representative immunofluorescence images of VE-cadherin (red) and CD31 (green) co-localization in colon tissues (nuclei counterstained with DAPI). (**B**) Left: Schematic of FITC-dextran (4 kDa) permeability assay. Right: Quantified serum FITC fluorescence intensity 60 min post-gavage (*n* = 5 per group). (**C**) Left: Schematic of Evans Blue vascular leakage assay. Right: Colonic Evans Blue extravasation quantified by absorbance at 620 nm (mean ± SD; *n* = 5 per group; ** *p* < 0.01, *** *p* < 0.001, ns: not significant; one-way ANOVA with Tukey’s test). (**D**) KEGG pathway analysis of RNA-seq data from colon tissues (DSS + BMP9 groups vs. DSS). (**E**) Volcano plot of differentially expressed genes. Genes highlighted in bold are key IBD-associated downregulated factors.

Following the demonstration of BMP9 efficacy in alleviating colitis-associated phenotypes, we next examined its effects on vascular barrier integrity using permeability-related assessments. Immunofluorescence analysis showed that DSS-treated mice exhibited an apparent increase in CD31^+^ vascular structures compared with control mice, accompanied by a reduced degree of VE-cadherin and CD31 co-localization, consistent with disrupted endothelial junctional organization (Figure 4A). BMP9 administration did not noticeably alter the DSS-induced increase in CD31^+^ vascular density. In contrast, VE-cadherin localization at CD31^+^ endothelial junctions was largely restored in BMP9-treated mice, reaching a pattern comparable to that observed in control animals. Functional assessment of paracellular permeability via oral FITC-dextran (4 kDa) revealed a significant reduction in leakage from 10.2 ± 1.3-fold (DSS vs. Control; *p* < 0.001) to 2.1 ± 0.4-fold (DSS + BMP9 vs. Control; *p* < 0.001; Figure 4B). Similarly, transvascular macromolecular leakage evaluated by Evans blue extravasation decreased from 2.4 ± 0.2-fold (DSS vs. Control; *p* < 0.001) to 1.5 ± 0.3-fold (DSS + BMP9 vs. Control; *p* = 0.007).

Transcriptomic sequencing with KEGG pathway enrichment analysis of differentially expressed genes between DSS + BMP9 and DSS groups identified significant modulation of the Inflammatory Bowel Disease pathway (Figure 4D–E). Among the 14 IBD-associated genes differentially regulated, BMP9 treatment downregulated key immune-related genes, including MHC class II antigen presentation molecules (*H2-Aa*, *H2-Ab1*, *H2-Eb1*, *H2-Oa*, *H2-Ob*, *H2-DMa*, *H2-DMb1*, *H2-DMb2*), pro-inflammatory cytokines and their receptors (*Il6*, *Il21*, *Il21r*, *Ifng*), and Th1/Th17 signaling components (*Stat4*).

### 3.5. BMP9/ALK1 Regulates Neutrophil Migration & Angiogenesis

**Figure 5:**
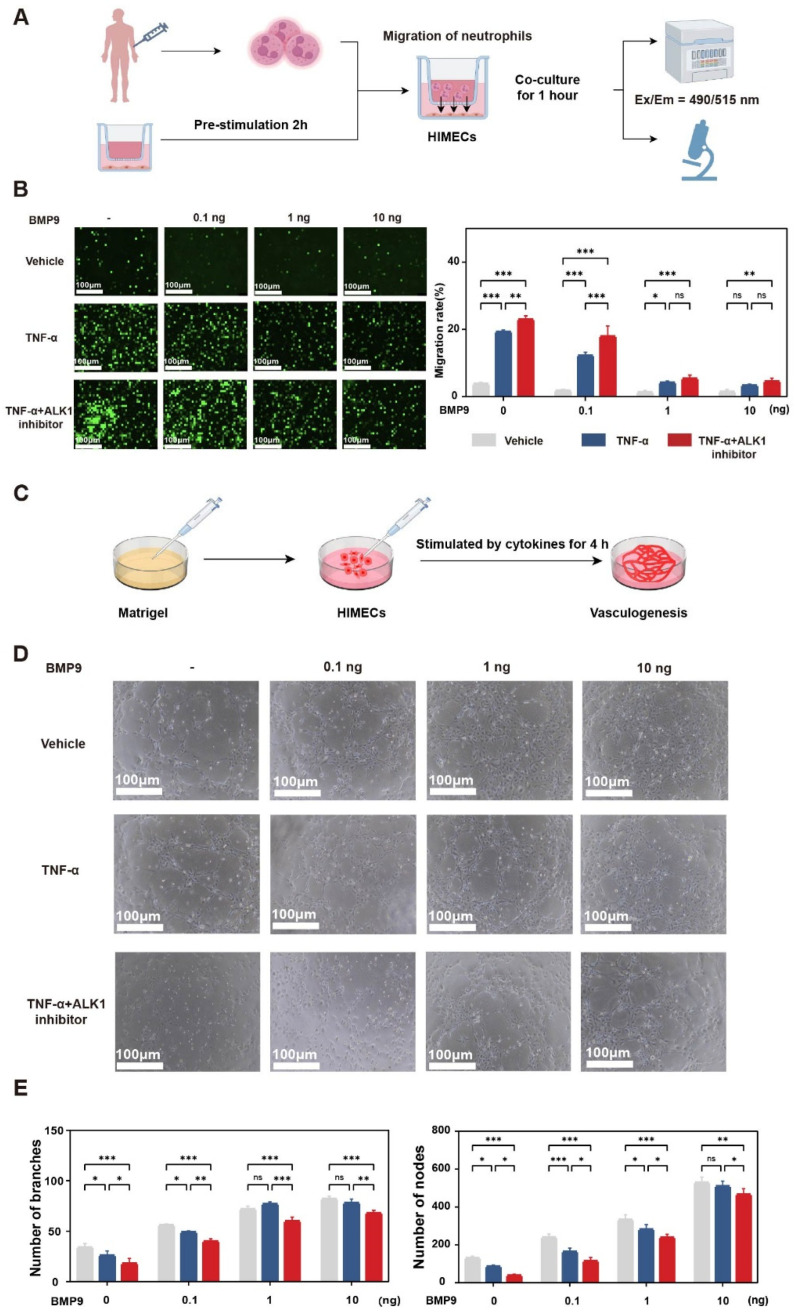
BMP9/ALK1 signaling modulates neutrophil migration and endothelial tube formation. (**A**) Schematic representation of the neutrophil migration assay. (**B**) (Left) Representative fluorescence micrographs demonstrating Calcein-AM-labeled neutrophil migration through 3 μm pore Transwell inserts toward HIMECs pretreated for 2 h with: vehicle control (0 ng/mL BMP9), BMP9 (0.1, 1, or 10 ng/mL), TNF-α (20 ng/mL as positive control), or ALK1 inhibitor ML347 (150 nM). (Right) Quantitative analysis of neutrophil migration rates (mean ± SD; * *p* < 0.05, ** *p* < 0.01, *** *p* < 0.001, ns: not significant by two-way ANOVA with post hoc testing). (**C**) Schematic illustration of the endothelial tube formation assay protocol. (**D**) Representative phase-contrast images of tubular network formation by HIMECs cultured on growth factor-reduced Matrigel under various treatment conditions: vehicle control (0 ng/mL BMP9), BMP9 (0.1, 1, or 10 ng/mL), TNF-α (20 ng/mL), or ML347 (150 nM). (**E**) Quantitative assessment of angiogenic parameters including branch points and nodal junctions (mean ± SD; * *p* < 0.05, ** *p* < 0.01, *** *p* < 0.001 by two-way ANOVA with appropriate post hoc comparisons).

Given our data demonstrating BMP9-mediated ALK1-Smad1 signaling activation, we hypothesized that impaired ALK1 signaling would exacerbate inflammatory infiltration and vascular leakage during colitis. To validate this, we established an in vitro migration model as depicted in Figure 5A: lower chambers containing TNF-α-stimulated HIMECs received TNF-α (20 ng/mL) with or without BMP9 pretreatment (0, 0.1, 1, or 10 ng/mL), while calcein-labeled human neutrophils were seeded in 3 μm upper chambers. Under basal conditions, spontaneous neutrophil migration was minimal and unaffected by BMP9. Upon TNF-α stimulation, neutrophil migration toward HIMECs was significantly enhanced. In contrast, BMP9 pretreatment dose-dependently suppressed TNF-α-induced migration, with maximal inhibition observed at 10 ng/mL. Crucially, the ALK1 antagonist ML347 (150 nM) abolished BMP9′s protective effect against TNF-α-induced migration (Figure 5B). Complementary endothelial tube formation assays revealed that TNF-α disrupted vascular networks, whereas BMP9 reversed this disruption and dose-dependently enhanced tube formation under non-inflammatory conditions. ML347 partially reversed BMP9-mediated enhancement of tube formation and stability (Figure 5C–E).

## 4. Discussion

From 1990 to 2019, China experienced a sustained 30-year rise in UC incidence, with a retrospective cohort study revealing a 42% 1-year refractory rate among Chinese UC patients following initial treatment [18]. While emerging evidence implicates gut vascular barrier (GVB) dysfunction as a potential driver of rUC progression [5], the precise molecular mechanisms underlying this pathological relationship remain poorly understood. In this study, we present multi-level evidence suggesting that dysregulation of the BMP9-ALK1 signaling axis may contribute to GVB impairment in rUC: clinical observations demonstrate that serum BMP9 deficiency and mucosal ALK1 downregulation are significantly associated with treatment-refractory status; experimental data show that BMP9 supplementation attenuates disease severity and restores GVB integrity in murine colitis; and mechanistic studies confirm that BMP9 enhances endothelial barrier function through ALK1-dependent signaling. These findings collectively highlight BMP9-ALK1 as a previously unrecognized pathogenic axis with potential therapeutic relevance.

In rUC patients, we identified a selective reduction in serum BMP9 levels—a defect not shared by its structural homolog BMP10—that significantly inversely correlated with post-treatment established disease activity indices (Modified Mayo Score, UCEIS). An important strength of our study design is that serum BMP9 was measured at baseline, prior to the initiation of advanced therapy, at which time the rUC and non-rUC groups exhibited comparable disease activity. This temporal relationship, combined with our stratified analyses demonstrating persistent BMP9 differences within disease severity strata, suggests that BMP9 deficiency may serve as a predictive biomarker for treatment response rather than simply reflecting disease severity. This systemic deficit corresponded with local colonic pathology: downregulated ALK1 expression, compromised VE-cadherin integrity, and overexpression of vascular adhesion molecules (VCAM-1/ICAM-1/MadCAM-1). Mechanistically, diminished VE-cadherin co-localization directly contributed to junctional defects underlying vascular hyperpermeability [19], while adhesion molecule upregulation facilitated leukocyte adhesion [20]. Notably, BMP9 administration in DSS-induced colitis attenuated these core pathological features, including GVB dysfunction (reduced small and macromolecular leakage), aberrant inflammation (decreased IL-1β and CCL2 levels), and early pro-fibrotic signaling (downregulated fibrogenic mediators such as α-SMA, TGFβ, Col1a1, and Col3a1), providing experimental support for the clinical observations.

Importantly, the protective effect of BMP9 was strictly dependent on ALK1 signaling, as pharmacological inhibition of ALK1 completely abolished the benefits conferred by BMP9, highlighting a target specificity that distinguishes it from other TGF-β ligands. Mechanistically, activation of the BMP9-ALK1 axis restored endothelial integrity through SMAD1/5-mediated reassembly of VE-cadherin and strongly suppressed neutrophil migration. Transcriptomic analysis provided further mechanistic insights into BMP9′s immunomodulatory actions. KEGG pathway enrichment revealed coordinated downregulation of IBD-associated genes following BMP9 treatment, encompassing three functionally interconnected modules. First, BMP9 suppressed multiple MHC class II molecules (H2-Aa, H2-Ab1, H2-Eb1, H2-Oa, H2-Ob, H2-DMa, H2-DMb1, H2-DMb2), which are essential for antigen presentation to CD4^+^ T cells and represent a critical checkpoint in adaptive immune activation during IBD [21]. Second, BMP9 downregulated key pro-inflammatory cytokines implicated in IBD pathogenesis, including IL-6 (a driver of acute-phase responses and Th17 differentiation), IL-21 (which amplifies Th17 responses and disrupts epithelial barrier function) [22], and IFN-γ (a canonical Th1 cytokine that promotes VE-cadherin disassembly) [23]. The concurrent suppression of Stat4, a transcription factor essential for Th1 cell differentiation and IFN-γ production [24], suggests that BMP9 may attenuate Th1-mediated inflammation at the transcriptional level. Third, the observed downregulation of IL-10—typically considered anti-inflammatory—likely reflects an overall dampening of the inflammatory milieu, as IL-10 production is often elevated in response to ongoing inflammation [25]. Collectively, these findings suggest that BMP9 exerts broad immunomodulatory effects by suppressing antigen presentation and T helper cell-associated inflammatory cascades, providing a mechanistic basis for its therapeutic efficacy in experimental colitis.

BMP9 was originally identified for its osteoinductive properties in bone and cartilage formation and participates broadly in embryonic development and cellular differentiation [26]. Our prior work established an inverse correlation between circulating BMP9 levels and non-alcoholic fatty liver disease (NAFLD) severity in metabolic syndrome (MetS) [27]. In that context, BMP9 was shown to ameliorate hepatic steatosis by modulating glucose/lipid metabolism, suppressing inflammation, and remodeling chromatin accessibility in high-fat diet (HFD) models [13]. Clinically, septic patients exhibit reduced serum BMP9, and in vitro studies demonstrate its capacity to enhance macrophage recruitment, phagocytosis, and bacterial clearance [28]. Our data, alongside existing literature, reveal a profound context-dependent functionality of BMP9 signaling. While BMP9 deficiency is now robustly linked to vascular destabilization–evidenced by loss of endothelial integrity in venous malformations, and aberrant vascular maturation in bmp9 KO zebrafish [29]—this pathway can paradoxically promote pathological angiogenesis in neoplasms [30]. This “double-edged sword” nature underscores the critical influence of the tissue microenvironment. This framework elegantly explains a key experimental observation: BMP9 treatment attenuated experimental colitis pathology without significantly reducing overall vascular density in DSS colitis, indicating its dominant therapeutic effect in this context is GVB stabilization, not broad anti-angiogenesis.

Long-standing active UC is associated with bowel wall fibrosis and vascular remodeling [31]. Although the acute DSS model used in our study does not recapitulate established fibrosis, we observed that BMP9 supplementation significantly attenuated expression of early pro-fibrotic markers (Collagen I/III, α-SMA, TGF-β), suggesting a potential role in limiting fibrogenic initiation. This contrasts with reports suggesting pro-fibrotic effects of supraphysiological BMP9 in hepatic models [32], while BMP9 deficiency may also contribute to hepatic fibrosis [33]. This apparent paradox emphasizes the tissue-specific regulation of BMP9 outcomes. We propose that in the intestinal microenvironment, the observed suppression of pro-fibrotic markers by BMP9 may result from ALK1 signaling dominance over profibrotic TGF-β responses within the endothelial niche; however, whether this translates to meaningful anti-fibrotic effects in chronic UC requires validation in long-term fibrosis models.

However, the systemic administration of BMP9 raises legitimate safety concerns given its well-documented osteoinductive properties. During our study, gross examination at necropsy revealed no evidence of ectopic calcification or heterotopic ossification in BMP9-treated mice. However, we acknowledge that the short treatment duration (7 days) may be insufficient to detect such complications. Future translational development would benefit from: (1) comprehensive evaluation of bone turnover markers and imaging surveillance in longer-term studies; (2) development of gut-targeted delivery systems to minimize systemic exposure. Colon-targeted nanoparticles or local injection during colonoscopy could enable localized BMP9 delivery to inflamed intestinal tissues while minimizing systemic circulation and potential osteoinductive effects [34].

Several limitations of our study should be acknowledged. First, the acute DSS model primarily induces epithelial injury and acute inflammation rather than the chronic, biologic-resistant phenotype characteristic of clinical rUC. While our study suggests the BMP9-ALK1 axis as a potential mechanistic link and provides proof-of-concept for therapeutic targeting, validation in chronic colitis models (e.g., T-cell transfer colitis, IL-10 knockout) would strengthen translational relevance. Second, although we demonstrated the crucial role of BMP9-ALK1 signaling in vascular barrier protection, its effects on other intestinal cell types (e.g., epithelial cells, fibroblasts) remain to be fully elucidated. Third, the systemic BMP9 administration approach used in this study warrants future development of gut-targeted delivery systems to enhance therapeutic efficacy while minimizing potential off-target effects. Fourth, the observational nature of human data precludes causal inference. Finally, the long-term therapeutic durability and disease-modifying potential of BMP9 treatment remain to be evaluated; therapeutic synergies with existing biologics like vedolizumab also remain unexplored.

Our findings carry several potential clinical implications. First, the strong inverse correlation between serum BMP9 levels and disease activity indices suggests BMP9 may serve as a non-invasive biomarker for monitoring disease severity and potentially predicting treatment response in UC patients. Second, the demonstration that BMP9 supplementation effectively restores GVB integrity even in the presence of persistent ALK1 downregulation indicates that residual receptor signaling capacity remains therapeutically targetable, offering a potential avenue for patients who have limited response to conventional biologic options. Third, the concurrent anti-inflammatory and barrier-protective effects of BMP9 suggest potential synergistic benefits when combined with existing therapies such as vedolizumab or anti-TNF agents.

## 5. Conclusions

In conclusion, our study suggests that dysregulation of the BMP9-ALK1 signaling axis may represent a significant, yet underappreciated, factor contributing to GVB dysfunction in ulcerative colitis. Our data indicate that systemic BMP9 deficiency is associated with treatment-refractory status and that restoring this signaling pathway attenuates experimental colitis severity by stabilizing endothelial junctions via ALK1. These findings highlight the potential of targeting endothelial integrity as a complementary strategy to current immune-centric therapies.

However, the translation of these findings to clinical practice warrants caution. The acute DSS model used in this study primarily recapitulates epithelial injury and inflammation, which may not fully reflect the complex, chronic pathology of human rUC. Additionally, the osteoinductive properties of BMP9 present potential safety challenges for systemic administration. Future research should therefore prioritize the validation of these mechanisms in chronic fibrosis models and the development of gut-targeted delivery systems to explore the therapeutic viability of this approach while minimizing off-target risks.

## Figures and Tables

**Table 1:** Baseline Characteristics of Study Participants.

Variable	Healthy Controls (*n* = 50)	Non-rUC (*n* = 47)	rUC (*n* = 48)	*p* Value
**Demographics**				
Age, years	40.4 ± 9.8	40.9 ± 12.1	39.7 ± 10.3	0.86
Sex, *n* (%)				0.89
Female	19 (38)	20 (42.6)	20 (41.7)	
Male	31 (62)	27 (57.4)	28 (58.3)	
BMI, Median (Q1, Q3)	23.7 (21.68, 25.9)	20.9 (19.35, 21.9)	20.35 (19.45, 21.85)	<0.001 †
Current smoker, *n* (%)	8 (16)	3 (6)	4 (8)	0.29
**Disease Characteristics**				
Disease extent, *n* (%)				0.35
E1 (Proctitis)	N/A	8 (17)	5 (10)	
E2 (Left-sided)	N/A	25 (53)	22 (46)	
E3 (Extensive)	N/A	14 (30)	21 (44)	
Modified Mayo Score, Median (Q1, Q3)	N/A	7 (5, 8.5)	8 (6, 9)	0.23
UCEIS, Median (Q1, Q3)	N/A	5 (4, 7)	6 (4, 7)	0.298
**Laboratory Parameters**				
CRP, mg/L, Median (Q1, Q3)	1.6 ± 1.5	33.5 ± 16.6	39.8 ± 14.3	<0.001 †
Hemoglobin, g/L, Mean ± SD	140 ± 12	116 ± 19	117 ± 18	<0.001 †
Albumin, g/L, Mean ± SD	44.4 ± 2.7	37.5 ± 3.6	36.9 ± 4.5	<0.001 †
Serum BMP9, ng/mL	289.85 ± 143.20	276.32 ± 122.77	181.42 ± 75.85	<0.001 ‡
**Concomitant Medications, *n* (%)**				
5-ASA	N/A	39 (83)	43 (90)	0.23
Corticosteroids	N/A	7 (15)	12 (25)	0.34
Immunomodulators	N/A	4 (9)	9 (19)	0.15
**Advanced Therapy History, *n* (%)**	N/A			
Anti-TNF	N/A	25 (53)	42 (88)	<0.001
Vedolizumab	N/A	12 (26)	37 (77)	<0.001
Ustekinumab	N/A	6 (13)	16 (33)	0.02
JAK inhibitors	N/A	4 (9)	25 (52)	<0.001
**Post-treatment Outcomes**	N/A			
Modified Mayo Score	N/A	4 (2, 5)	7 (5, 9)	<0.001
UCEIS	N/A	3 (1.5, 4.5)	5 (4, 7)	<0.001
Clinical response §, *n* (%)	N/A	47 (100)	0 (0)	<0.001

† Significant difference between healthy controls and UC groups; no significant difference between non-rUC and rUC. ‡ One-way ANOVA; post-hoc: HC vs. non-rUC *p* = 0.62, HC vs. rUC *p* < 0.001, non-rUC vs. rUC *p* < 0.001. § Clinical response defined as Modified Mayo Score reduction ≥3 points after ≥14 weeks of advanced therapy. Abbreviations: rUC, refractory ulcerative colitis; BMI, body mass index; UCEIS, Ulcerative Colitis Endoscopic Index of Severity; CRP, C-reactive protein; 5-ASA, 5-aminosalicylic acid; TNF, tumor necrosis factor; JAK, Janus kinase; BMP9, bone morphogenetic protein 9; N/A, not applicable.

## Data Availability

All datasets supporting the conclusions of this article are included within the manuscript and Supplementary Materials. Raw clinical data are subject to institutional patient confidentiality provisions and are therefore not deposited in public repositories. Non-identifiable datasets generated during this study are available from the corresponding author upon fulfillment of institutional data transfer agreements for academic research purposes.

## Supplementary Materials

The following supporting information can be downloaded at: https://www.mdpi.com/article/10.3390/biomedicines14020288/s1, Table S1: Primer Sequences for RT-qPCR. Table S2: Serum BMP9 Levels Stratified by Baseline Disease Severity.
