# Supplementary material for: Recombinant BMP9 Reinforces Gut Vascular Barrier in Experimental Colitis
Source: Biomedicines. 2026 Jan 28;14(2):288. doi: 10.3390/biomedicines14020288 (PMC12937769; doi:10.3390/biomedicines14020288)
Supplement: Supplementary file 1 [file biomedicines-14-00288-s001.zip › Supplementary Table S1.pdf]

Supplementary Table S1. Primer Sequences for RT-qPCR

**Gene**	**5'-3'**
H-GAPDH-F	GGAGCGAGATCCCTCCAAAAT
H-GAPDH-R	GGCTGTTGTCATACTTCTCATGG
H-ALK1-F	CGAGGGATGAACAGTCCTGG
H-ALK1-R	GTCATGTCTGAGGCGATGAAG
H-IL6-F	ACTCACCTCTTCAGAACGAATTG
H-IL6-R	CCATCTTTGGAAGGTTCAGGTTG
H-TNFα-F	CCTCTCTCTAATCAGCCCTCTG
H-TNFα-R	GAGGACCTGGGAGTAGATGAG
H-CCL2-F	CAGCCAGATGCAATCAATGCC
H-CCL2-R	TGGAATCCTGAACCCACTTCT
H-CD31-F	AACAGTGTTGACATGAAGAGCC
H-CD31-R	TGTAAAACAGCACGTCATCCTT
H-ICAM1-F	ATGCCCAGACATCTGTGTCC
H-ICAM1-R	GGGGTCTCTATGCCCAACAA
H-VCAM1-F	GGGAAGATGGTCGTGATCCTT
H-VCAM1-R	TCTGGGGTGGTCTCGATTTTA
H-MadCAM1-F	GGGAGAAGTGATCCCAACAGG
H-MadCAM1-R	CGTTTCCAGAGGTGATACGTG
M-Gapdh-F	AGGTCGGTGTGAACGGATTTG
M-Gapdh-R	TGTAGACCATGTAGTTGAGGTCA
M-Ccl2-F	TTAAAAACCTGGATCGGAACCAA
M-Ccl2-R	GCATTAGCTTCAGATTTACGGGT
M- Il1β-F	GAAATGCCACCTTTTGACAGTG
M-Il1β-R	TGGATGCTCTCATCAGGACAG
M-Col1a1-F	GGAGCCTGAGTCAGCAGATTG
M-Col1a1-R	AGGTTGCAGCCTTGGTTAGG
M-Alk1-F	CTGGGTGCTCTAGGCTTGTG
M-Alk1-R	GCCCGTAGTACAGTCGCTG
M-Col3a1-F	CATGACTGTCCCACGTAAGCA
M-Col3a1-R	TGGTATGTAATGTTCTGGGAGGC
