# Supplementary material for: Recombinant BMP9 Reinforces Gut Vascular Barrier in Experimental Colitis
Source: Biomedicines. 2026 Jan 28;14(2):288. doi: 10.3390/biomedicines14020288 (PMC12937769; doi:10.3390/biomedicines14020288)
Supplement: Supplementary file 1 [file biomedicines-14-00288-s001.zip › Supplementary Table S2.pdf]

Supplementary Table S2. Serum BMP9 Levels Stratified by Baseline Disease Severity

Baseline Disease Severity*	Non-rUC	rUC	*P* value
**Mild (Mayo 3–5)**			
N	13	7	—
Serum BMP9, ng/mL	317.44 ± 106.37	242.37 ± 119.36	0.191
**Moderate (Mayo 6–10)**			
N	34	40	—
Serum BMP9, ng/mL	260.60 ± 126.40	172.52 ± 62.18	< 0.001
**Severe (Mayo 11–12)**			
N	0	1	—
Serum BMP9, ng/mL	—	110.91	N/A
**Overall**			
N	47	48	—
Serum BMP9, ng/mL	276.32 ± 122.77	181.42 ± 75.85	< 0.001

*Disease severity was categorized according to the Modified Mayo Score at baseline (pre-treatment): mild (3–5), moderate (6–10), severe (11–12).
P values were calculated using independent t-test.
Abbreviations: rUC, refractory ulcerative colitis; BMP9, bone morphogenetic protein 9; N/A, not applicable.
